# β-Conglycinin Reduces the Tight Junction Occludin and ZO-1 Expression in IPEC-J2

**DOI:** 10.3390/ijms15021915

**Published:** 2014-01-27

**Authors:** Yuan Zhao, Guixin Qin, Rui Han, Jun Wang, Xiaodong Zhang, Dandan Liu

**Affiliations:** 1 College of Animal Science and Technology, Jilin Agricultural University, Changchun 130118, China; E-Mails: zhaoyuan4CL52@126.com (Y.Z.); hanrui0409@163.com (R.H.); wangjun4169@126.com (J.W.); ddliu19880922@163.com (D.L.); 2 Key Laboratory of Animal Production, Product Quality and Security, Ministry of Education, Jilin Agricultural University, Changchun 130118, China; 3 Key Laboratory of Zoonosis Research, Ministry of Education, Institute of Zoonosis, College of Veterinary Medicine, Jilin University, Changchun 130062, China; E-Mail: zhang_xd@jlu.edu.cn

**Keywords:** β-conglycinin, intestinal barrier, tight junction, food allergen, IPEC-J2

## Abstract

Soybean allergy presents a health threat to humans and animals. The mechanism by which food/feed allergen β-conglycinin injures the intestinal barrier has not been well understood. In this study, the changes of epithelial permeability, integrity, metabolic activity, the tight junction (TJ) distribution and expression induced by β-conglycinin were evaluated using IPEC-J2 model. The results showed a significant decrease of trans-epithelial electrical resistance (TEER) (*p* < 0.001) and metabolic activity (*p* < 0.001) and a remarkable increase of alkaline phosphatase (AP) activity (*p* < 0.001) in a dose-dependent manner. The expression levels of tight junction occludin and ZO-1 were decreased (*p* < 0.05). The reduced fluorescence of targets and change of cellular morphology were recorded. The tight junction occludin and ZO-1 mRNA expression linearly declined with increasing β-conglycinin (*p* < 0.001).

## 1. Introduction

The increasing consumption of various soybean products owing to their high nutritional value has lead to a rise in the incidence of soybean allergies [1]. Soybean β-conglycinin, the major food/feed allergen, is recognized by IgE antibodies present in soybean-allergic humans [2,3] and animals [4].

The intestinal mucosa represents a crucial border between the organism and its environment. The large contact surface allows efficient nutrient absorption, acts as an important barrier for pathogens and toxins, and participates in the innate immune response [5,6]. As recently reported, β-conglycinin may produce three major enzyme-hydrolyzed peptides (the molecular weight of 52, 30 and 25 kD, respectively) with the intact IgG and IgE binding epitopes after digestion by continuous pepsin and trypsin [7]. Both digested and undigested β-conglycinin can transit through the stomach and small intestine [8–11], and be absorbed in immunoreactive form by the gut epithelium, facilitating the exposition of these allergens to the immune system, which would consequently elicit an allergic response in a sensitized individual [12,13].

Piglets easily suffer from soybean-induced allergy, so the presence of allergenic components including intact β-conglycinin and its enzyme-hydrolyzed peptides greatly restricts their use in neonatal and weanling pigs. Soybean allergies usually cause inflammatory disorders in the small intestine, characterized by villous atrophy and hyperplasia in the crypt, as well as accelerated enterocyte proliferation, apoptosis and migration [14–18]. However, so far, there have been few studies into the effects of β-conglycinin on the intestinal barrier permeability of piglets.

The intestinal porcine epithelial cells originated from jejunum (IPEC-J2) retained most of their original epithelial nature [5]. So we opted for the use of an IPEC-J2 that would maximally resemble the *in vivo* situation. Meanwhile, it could also give some related hints for soybean-allergic patients due to porcine physiological and immunological similarity to human beings [19,20]. In this study, the epithelial permeability, integrity, metabolic activity, tight junction (TJ) distribution and expression in IPEC-J2 respectively treated with β-conglycinin were determined, which are essential criteria to explore the mechanism of soybean-induced allergy.

## 2. Results

### 2.1. TEER

**Figure 1.**
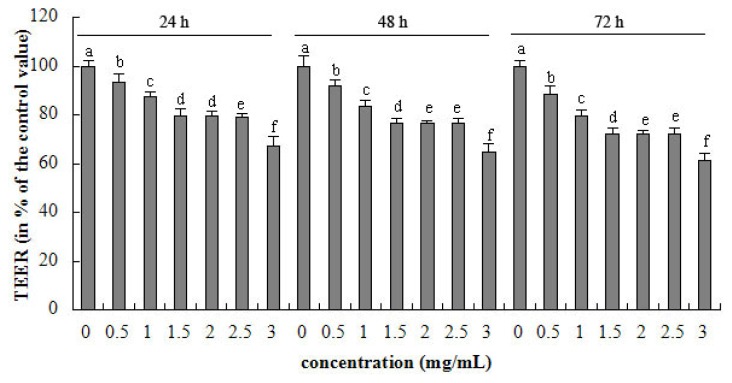
Trans-epithelial electrical resistance (TEER) of intestinal porcine epithelial cells originated from jejunum (IPEC-J2) cells after 24, 48 and 72 h incubation of β-conglycinin. Each bar represents four independent experiments performed in triplicates ± SD. a, b, c, d, e, f each represent two groups for different concentrations which are statistically different from each other.

As shown from Figure 1, 0–3 mg/mL β-conglycinin decreased the TEER after 24, 48 and 72 h incubation in a dose-dependent manner. The negative linear correlation existed between TEER and β-conglycinin (*p* < 0.001, *R*^2^ = 0.919) levels at 24 h. The highest concentration of β-conglycinin (3 mg/mL) reduced the TEER to 67% of the control after 24 h, respectively. The maximal reduction of TEER (*versus* control) was 38% after 72 h incubation, respectively.

### 2.2. Cellular Metabolic Activity Detected by MTT Assay

**Figure 2.**
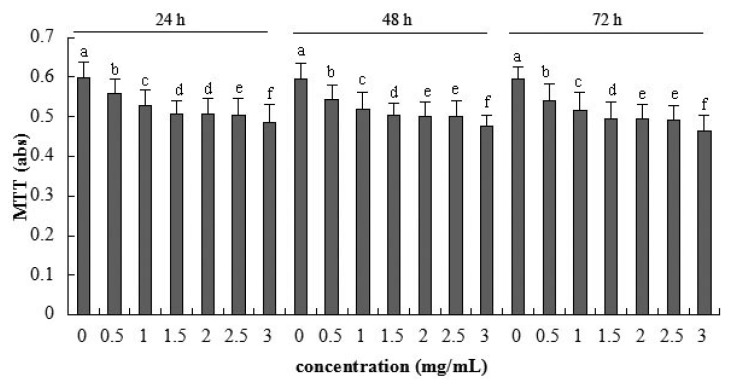
Metabolic activity of IPEC-J2 cells after 24, 48 and 72 h incubation of β-conglycinin measured by MTT assay. Each bar represents four independent experiments performed in triplicates ± SD. a, b, c, d, e, f each represent two groups for different concentrations which are statistically different from each other.

A significant linear reduction of cellular metabolic activity was detected after application of 0.5–3 mg/mL β-conglycinin (*R*^2^ = 0.862, *p* < 0.001) at 24 h (Figure 2). The highest β-conglycinin concentration (3 mg/mL) reduced the metabolic activity to the minimum 78% (*versus* control) after 72 h incubation, respectively.

### 2.3. Cellular Integrity Assessed by AP Activity

**Figure 3.**
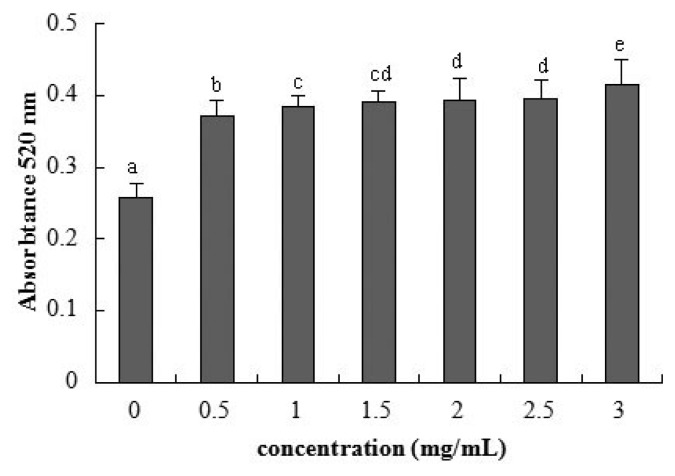
Alkaline phosphatase activity of IPEC-J2 cells after 72 h incubation of β-conglycinin. Each bar represents four independent experiments performed in triplicates ± SD. a, b, c, cd, d, e each represent two groups for different concentrations which are statistically different from each other.

Detection of AP activity as a marker for enterocyte differentiation [21] showed a remarkable increase for 0.5 mg/mL β-conglycinin at 72 h (Figure 3). The AP activity had a positive linear relationship with the β-conglycinin levels (*R*^2^ = 0.603, *p* < 0.001).

### 2.4. Tight Junction Distribution and Expression

**Figure 4.**
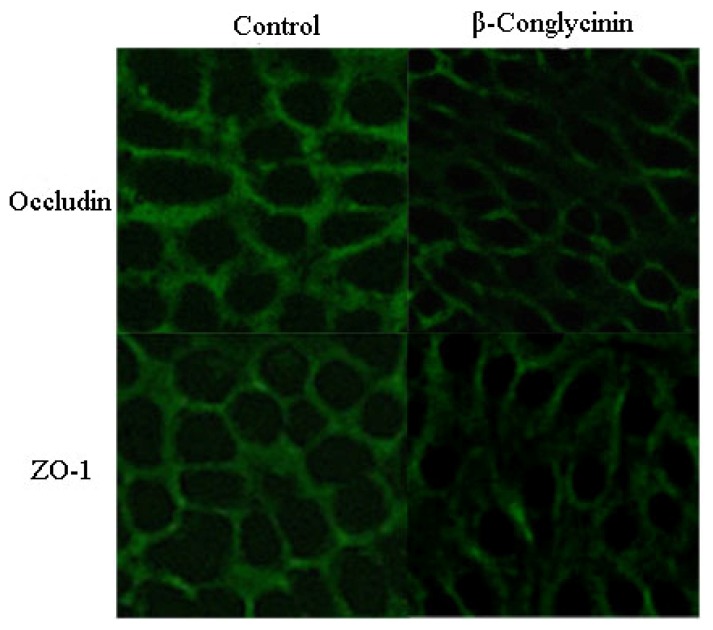
Tight junction proteins distribution of IPEC-J2 cells after 24 h incubation of β-conglycinin. Representative images of immunofluorescence staining (magnification 200×) are shown.

**Figure 5.**
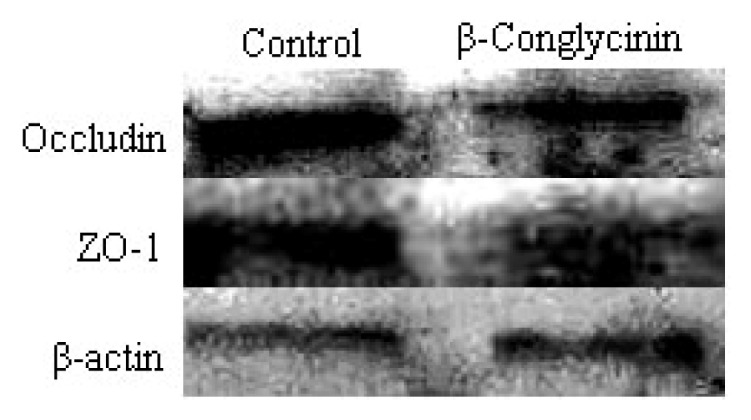
Western blot of tight junction proteins of IPEC-J2 cells after 24 h incubation of β-conglycinin. The β-actin is housekeeping protein. Representative western blots from four independent experiments are shown.

The tight junction proteins (occludin and zonula occluden (ZO)-1) were located at the cell-cell contact regions, as shown in Figure 4. After treatment with β-conglycinin, the cellular morphology was altered, and the cellular junction location was obscure. The staining intensity of occludin and ZO-1 clearly decreased compared with the control. The protein expression of occludin and ZO-1 were clearly reduced by 46% and 15% when analyzed by western-blot (Figure 5), respectively (*p* < 0.05).

### 2.5. Tight Junction mRNA Expression

**Figure 6.**
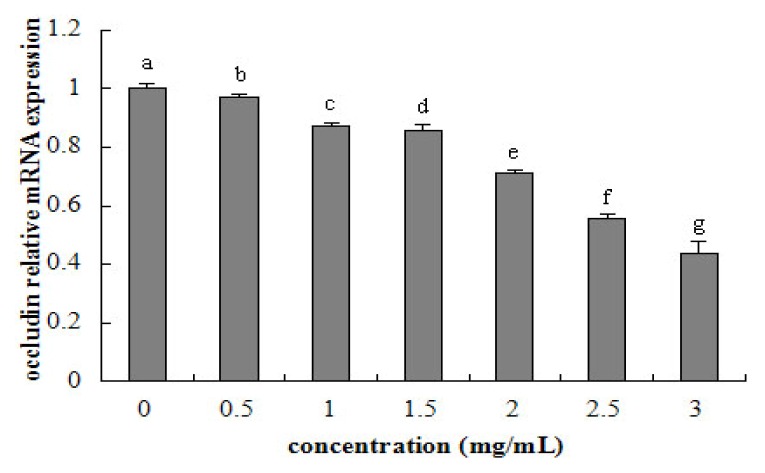
Occludin relative mRNA expression of IPEC-J2 cells after 24 h incubation of β-conglycinin. Each bar represents four independent experiments performed in triplicates ± SD. a, b, c, d, e, f, g each represent two groups for different concentrations which are statistically different from each other.

**Figure 7.**
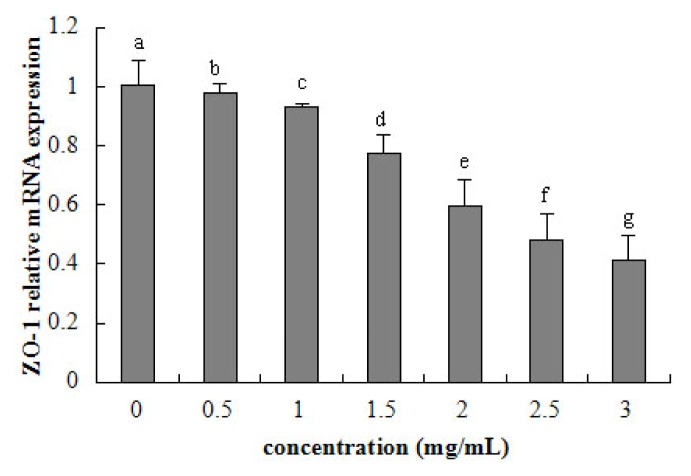
ZO-1 relative mRNA expression of IPEC-J2 cells after 24 h incubation of β-conglycinin. Each bar represents four independent experiments performed in triplicates ± SD. a, b, c, d, e, f, g each represent two groups for different concentrations which are statistically different from each other.

Tight junction occludin or ZO-1 mRNA expression was assessed after 24 h exposure to β-conglycinin of different levels. The tight junction mRNA expression tended to linearly decline with increasing β-conglycinin (occludin: *R*^2^ = 0.941, *p* < 0.001; ZO-1, *R*^2^ = 0.956, *p* < 0.001) from 0.5–3 mg/mL (Figures 6 and 7). After 24 h incubation, the maximal reduction of occludin (*versus* control) and ZO-1 (*versus* control) was 57% and 59% for β-conglycinin.

## 3. Discussion

Food allergy is an increasing clinical problem and has been estimated to affect 5%–6% of children and 3%–4% of the adult population [22,23]. Egg ovalbumin, cow milk, wheat, peanuts, and soybean allergic proteins are commonly known as food allergens [24]. The intestinal epithelium has not only an absorption function for dietary factors but also a barrier function to restrict the permeation of noxious substances. Physiologically, most dietary proteins are digested to small peptides or amino acids, which are then absorbed into enterocytes. A small amount of intact allergic protein may be endocytosed into epithelial cells for degradation and lose their antigenic properties [25–28]. In food allergy, IgE/CD23 mediate antigen transcellular transport in the epithelial cells prior to mast cell activation, or it crosses epithelial cells through the paracellular pathway and transcellular pathway after the release of mast cell mediators [29,30].

Food allergy such as induced by ovalbumin (OVA) could increase the intestinal permeability [31–34]. TEER is used as a parameter of epithelial barrier function, which indicates variation of integrity and permeability of the cell monolayer [35,36]. The present study displays that β-conglycinin induced low TEER of intestinal epithelium. This means that the soybean allergen can enhance the epithelial permeability. TEER measure is usual in toxicology but rare in food allergy. Results regarding the wheat germ agglutinin toxin are accordance with our results [37].

The MTT assay is a standard method for the detection of metabolic activity in cell culture, which indirectly demonstrates the damage of membrane function. The present data show that β-conglycinin reduces the cellular metabolic activity in a dose-dependent manner, suggesting the reduction of cellular activity, which is similar to Xu *et al*. (2010) [38] using the mouse intestinal epithelial cells as model. Transient hypersensitivity to soybean antigens could lead to villus atrophy, crypt hypertrophy [14–16,39]. In addition, β-conglycinin may accelerate the enterocyte apoptosis and the relative enterocyte migration rate for piglets in our previous study [18]. The above literature provides evidence that β-conglycinin could induce the low enterocyte activity.

Intestinal AP has an essential function in maintaining epithelial integrity, whose loss may increase the permeability to inflammation and sepsis [40,41]. In our present study, the experimental results show that the AP activity showed a remarkable increase, so β-conglycinin has significant negative effects on the cellular integrity. This corresponds to the changed epithelial permeability.

TJ, a major mode of cell-to-cell adhesion, is a key determinant of intestinal barrier function preventing bacteria, endotoxin and toxicity macromolecule from crossing an epithelial sheet between adjacent cells. It is organized by specific interactions between various intracellular proteins (ZO-1, ZO-2, ZO-3, MUPP-1) and transmembrane proteins (occludin, claudin, and junctional adhesion molecules) [42]. The reduction of occludin [43] and ZO-1 [44] can enhance the epithelial permeability in a number of cell systems. After a challenge with food allergen, the increased permeability has been shown to initially amplify allergen ingestion and translocation through the transcellular route, and subsequently enlarge the paracellular permeability associated with a disruption of TJ after sensitization depending on activated mast cells [29,45]. However, literature regarding soybean allergen and epithelial integrity and permeability are rare. It has been found in our study that the β-conglycinin decreased occludin and ZO-1 in IPEC-J2, which also demonstrates that the soybean β-conglycinin destroys the epithelial barrier owing to the reduced TJ expression.

The regression analysis suggests that β-conglycinin has a linear relationship with the cellular permeability, metabolic activity, integrity, and TJ gene expression. This means that the increasing soybean allergens induce the tight junction to loosen, and then augment the epithelial permeability. Moreover, from observation, it seems that indexes of the current study change seriously with the extension of incubation time.

## 4. Methods and Materials

### 4.1. Preparation of β-Conglycinin

β-Conglycinin was isolated from defatted soy flour by the method of Setsuko *et al*. (1987) [46]. The globulins sample contained over 95% β-conglycinin determined by the Kjeldahl method and SDS-PAGE analysis.

### 4.2. Cells and Culture Conditions

The porcine intestinal epithelial cells from jejunum (IPEC-J2) were kindly donated by Guoyao Wu of China Agricultural University. The IPEC-J2 were seeded in cell culture flasks and cultured in DMEM/F12 medium (Gibco, Carlsbad, NM, USA), supplemented with 10% fetal bovine serum (FBS, Gibco, Carlsbad, NM, USA), 1% Penicillin-Streptomycin (Sigma, St. Louis, MO, USA), and 1% glutamine (Amersco, Solon, Tucson, AZ, USA) at 37 ºC in a humidified atmosphere of 5% CO_2_ (Selecta, Barcelona, Spain). The culture medium was changed every other day.

### 4.3. Trans-Epithelial Electrical Resistance (TEER) Measurement

IPEC-J2 were seeded on the millicell membrance (Millipore, Billerica, MA, USA, 0.3 μm pore size) cell culture inserts (Costar, Corning Inc., New York, NY, USA) at a density of 5 × 10^4^/cm^2^. When monolayer of cells was completely differentiated, cells were treated with 0, 0.5, 1.0, 1.5, 2.0, 2.5, 3.0 mg/mL of β-conglycinin. The TEER was measured using the millicell-ERS resistance system (Millipore, Billerica, MA, USA) after incubation for 24, 48, and 72 h, respectively.

### 4.4. MTT Assay

Metabolic activity was measured by MTT assay. IPEC-J2 were then treated with 0, 0.5, 1.0, 1.5, 2.0, 2.5, 3.0 mg/mL β-conglycinin for 24, 48, and 72 h, respectively. Exactly 20 μL of MTT solutions (Sigma, Aldrich Inc., St. Louis, MO, USA) was added to each well and incubated for 4 h at 37 ºC. The mixture reaction was carefully taken out and 150 μL dimethyl sulfoxide (DMSO, Solarbio, Shanghai, China) was added to each well, which was allowed to stand 10 min to allow it to completely mix. After the crystals were fully dissolved, the optical density was measured at 570 nm by Microplate Reader (Bio-Rad, Hercules, CA, USA).

### 4.5. Alkaline Phosphatase Activity Assay

Cellular membrane integrity was assessed by measurement of alkaline phosphatase (AP) activity in the supernatant. Cells were grown in a 96-well microplate and treated with 0, 0.5, 1.0, 1.5, 2.0, 2.5, 3.0 mg/mL β-conglycinin for 72 h, respectively. The AP activity of the incubation medium was measured by the AP Test kit (Jiancheng, Nanjing, China).

### 4.6. Analysis of Tight Junction Proteins Structure by Immunofluorescence

IPEC-J2, grown on cover glass within six well plates (Nest, Beijing, China) for complete confluence, were treated with 0.5 mg/mL β-conglycinin media for 24 h. After rinsing in PBS three times, samples were fixed with cold acetone for 15 min at room temperature. Next, cells were permeabilized with 0.5% Triton X-100 for 5 min and blocked with 5% Bovine Serum Albumin (BSA) for 30 min at 37 ºC. After washing, samples were incubated with rabbit-anti-occludin or rabbit-anti-ZO-1 (diluted 1:100 or 1:100 in 1% of BSA respectively, Bios, Beijing, China) as the primary antibody for 30 min at 37 ºC then with FITC-conjugated goat-anti-rabbit (diluted 1:50, CWBIO, Beijing, China) as the second antibody for 2 h at room temperature. The views for each sample were captured using laser scanning confocal microscope (Nikon, Tokyo, Japan) and representative images were presented.

### 4.7. Western Blot Analysis

IPEC seeded in cell culture flasks were treated with 0.5 mg/mL β-conglycinin media for 24 h, then cells were washed with PBS (0.01 M). Every flask of samples was lysed on ice with a 500 μL cell lysis buffer (20 mM Tris-HCL pH 8.0, 5 mM EDTA and 1% Triton X-100) and supplemented with protease inhibitors (Cat. 539134, Merck, Darmstadt, Germany) for 30 min. Sample collections were sonicated three times for 20 s and centrifuged at 10,000× *g* for 30 min at 4 ºC. Protein quantification of supernatant was determined by method of BCA kit (Thermo, Waltham, MA, USA).

Exactly 12 μg of cell total proteins extraction was separated by SDS-PAGE and transferred onto PVDF membranes. The rabbit anti-occludin, rabbit anti-ZO-1 or rabbit anti-β-actin (Bios, Beijing, China) antibodies were used as the primary antibody. The horseradish peroxidase-conjugated anti-rabbilt IgG (diluted 1:3000, Tianjin Sungene Biotech Co., Tianjin, China) was incubated as the second antibody. The Luminata Crescendo Western HRP Substrate (Millipore, Billerica, MA, USA) was applied for chemiluminescent detection using tabletting and autoradiographic film (Kodak, Xiamen, China). Grey levels were estimated using Quantity One software-4.6.2 (Basic) (Bio-Rad, Hercules, CA, USA). Western blot band density was compared to β-actin in each lane as a loading control.

### 4.8. RNA Extraction

The IPEC-J2 was stimulated with varying concentrations of β-conglycinin (0, 0.5, 1, 1.5, 2, 2.5, 3.0 mg/mL) for 24 h. Then the cells were rinsed twice with phosphate-buffered saline (PBS) without Ca^2+^ and Mg^2+^, harvested, centrifuged, and subjected to RNA extraction procedure.

Total cellular RNA was extracted from IPEC-J2 with E.Z.N.A. Total RNA Kit II (Omega, Doraville, GA, USA) according to the manufacturer’s instructions. To prevent DNA contamination, DNase digestion was performed with RNase-free DNase. The Purified RNA extracts were stored at −80 ºC in RNase, DNase-free water (Takara, Otsu, Japan) until use. The concentration and purity of RNA extracts was determined using the absorbance at 260 and 280 nm in a spectrophotometer (Beckmandu-800, Fullerton, CA, USA). The samples with the ratio 260 nm/280 nm between 1.9 and 2.1 were further evaluated for their quality. The integrity of RNA was determined on 1% agarose gels and visualization of the 28S and 18S ribosomal RNA.

### 4.9. Reverse Transcription

Reverse transcription was performed using the High Capacity cDNA Reverse Transcription Kits (Applied Biosystems, Foster City, CA, USA). The reverse transcription was carried out in a 20 μL final volume that included 2 μL 10× RT Buffer, 0.8 μL 25× dNTP Mix (100 mM), 2 μL 10× RT Random Primers, 1 μL MultiScribe™ Reverse Transcriptase (Applied Biosystems, Foster City, CA, USA), 2 μg RNA template, and Nuclease-free H_2_O to complete the final volume. The reserve transcription mix was incubated at 25 ºC for 10 min, heated to 37 ºC for 120 min, and finally inactivated at 85 ºC for 5 min. The resultant cDNA was stored at −80 ºC until use.

### 4.10. Real-Time Quantitative PCR Analysis

The real-time quantitative PCR was carried out using a StepOne Plus real-time PCR system (Applied Biosystems, Foster City, CA, USA). TaqMan Gene Expression Master Mix was used according to manufacturer’s specifications (Applied Biosystems, Foster City, CA, USA). Primer and probe sets for target genes were prevalidated in TaqMan Gene Expression Assay kits (ABI): (ZO-1 Assay ID: Ss03373514; Occludin Assay ID: Ss03377507; β-actin Assay ID: Ss03376081). The PCR reaction was carried out in 96-well reaction plates with 10 μL TaqMan Gene Expression Master Mix (2×), 1 μL TaqMan Gene Expression Assay (20×), 2 μL cDNA template and 7 μL Nuclease-free H_2_O. The 40 thermal cycles of 2 min at 50 ºC, 10 min at 95 ºC, 15 s at 95 ºC and 1 min at 60 ºC were utilized according to manufacturer recommendations.

Relative quantification of target genes was calculated using the 2^−ΔΔ^*^C^*^t^ method. Target gene expression was normalized to β-actin messenger RNA (mRNA) levels.

### 4.11. Statistical Analysis

Data of relative gene expression were analyzed using the general linear model procedure of Statistical Package for Social Sciences version 11.5 (SPSS Inc., Chicago, IL, USA). Differences among means were tested using Duncan’s multiple range tests. Linear regression with calculation of the correlation factor (*r*) was used for jointly distribution variables. Statements of statistical significance were based upon *p* ≤ 0.05.

## 5. Conclusion

The altered TJ expression induced by β-conglycinin gives rise to the barrier dysfunction of intestinal epithelium.
